# To Acquire or Not to Acquire: Evaluating Compressive Sensing for Raman Spectroscopy in Biology

**DOI:** 10.1021/acssensors.4c01732

**Published:** 2024-12-20

**Authors:** Piyush Raj, Lintong Wu, Jeong Hee Kim, Raj Bhatt, Kristine Glunde, Ishan Barman

**Affiliations:** † Department of Mechanical Engineering, Johns Hopkins University, Baltimore, Maryland 21218, United States; ‡ Hackensack Meridian School of Medicine, Nutley, New Jersey 07110, United States; § The Russell H. Morgan Department of Radiology and Radiological Science,The Johns Hopkins University, School of Medicine, Baltimore, Maryland 21205, United States; ∥ Department of Biological Chemistry, The Johns Hopkins University School of Medicine, Baltimore, Maryland 21205, United States; ⊥ The Sidney Kimmel Comprehensive Cancer Center, The Johns Hopkins University School of Medicine, Baltimore, Maryland 21287, United States

**Keywords:** Raman spectroscopy, compressive sensing, sparse data, chemical biology, miniaturization

## Abstract

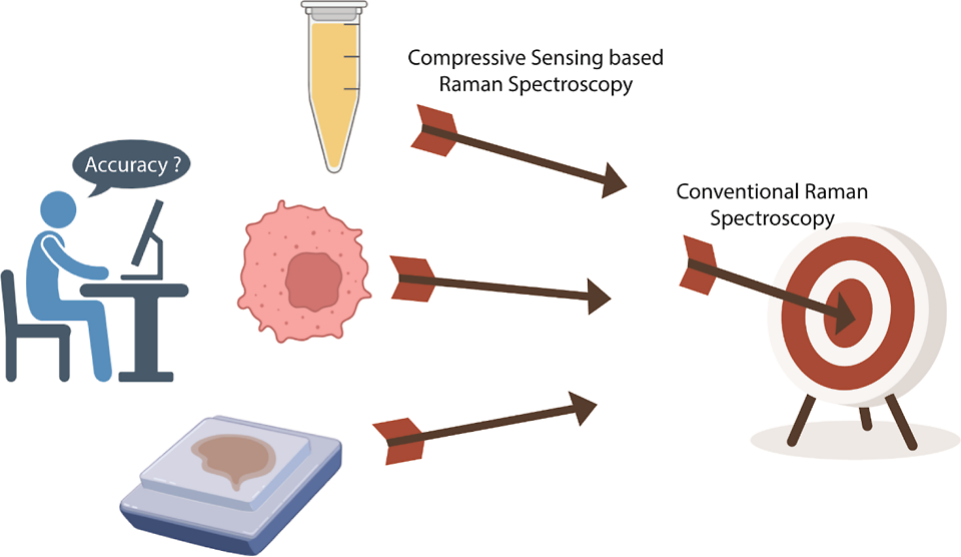

Raman spectroscopy has revolutionized the field of chemical biology by providing detailed chemical and compositional information with minimal sample preparation. Despite its advantages, the technique suffers from low throughput due to the weak Raman effect, necessitating long acquisition times and expensive equipment. This limitation is particularly acute in time-sensitive applications like bioprocess monitoring and dynamic studies. Compressive sensing offers a promising solution by reducing the burden on measurement hardware, lowering costs, and decreasing measurement times. It allows for the collection of sparse data, which can be computationally reconstructed later. This paper explores the practical application of compressive sensing in spontaneous Raman spectroscopy across various biological samples. We demonstrate its benefits in scenarios requiring portable hardware, rapid acquisition, and minimal storage, such as skin hydration prediction and cellular studies involving drug molecules. Our findings highlight the potential of compressive sensing to overcome traditional limitations of Raman spectroscopy, paving the way for broader adoption in biological research and clinical diagnostics.

Raman spectroscopy has become a pivotal tool in the field of chemical biology over the last two decades.^1^ Its ability to provide detailed chemical and compositional information in a label-free manner, with minimal sample preparation and high multiplexing capability, has made it indispensable for studying biomolecules, cells, tissues, and even entire organisms at the molecular level.^2−7^ This technique holds significant promise for various biological applications, including disease diagnosis, drug development, and understanding cellular processes.^3,8,9^ The COVID-19 pandemic recently acted as a catalyst for developing Raman-based applications for disease detection, highlighting Raman spectroscopy’s versatility and rapid deployment potential.^10,11^

Raman spectroscopy works on the principle of inelastic scattering where a photon, usually in the UV, Vis or the NIR region interacts with the molecule resulting in a shift in energy corresponding to the molecular vibration. This energy shift indicates the presence of discrete vibrational modes of molecules, thereby revealing biochemical composition information. When the instrument response is appropriately calibrated, the intensity of the signal gives a quantitative measurement of the composition.^12^ Furthermore, Raman microscopes allow us to collect spatially resolved molecular distributions in cells and tissues, making it a powerful approach in biological research. However, the inherent limitation of Raman spectroscopy lies in its low throughput due to the weak Raman effect.^13^ Most biomolecules have small Raman cross sections^14^ requiring long acquisition times and costly sensitive detectors and spectrometers for signal collection. This issue is exacerbated in time-sensitive applications such as bioprocess monitoring, rapid dynamic studies, and scenarios sensitive to light dosage.

To address these challenges, significant efforts have been made to enhance the throughput of Raman measurements. One approach to accelerating the measurement process involves data-driven acquisition techniques, where real-time information gathered during the measurement can dynamically guide the sampling strategy. By analyzing the data as it is being collected, the system can identify regions or subsets of the data set that are more informative or contain critical features, allowing the algorithm to focus on those areas for further sampling.^15,16^ Innovative hardware solutions like line-scanning Raman systems^17^ enable rapid collection of spectra from large sample areas, facilitating real-time monitoring of dynamic processes. Despite these advancements, these systems tend to be more expensive, complex, and bulkier. Additionally, the vast data generated in hyperspectral measurements poses substantial challenges in terms of storage, retrieval, analysis, and archiving.

Compressive sensing may provide a transformative solution for these issues.^18^ By reducing the burden on measurement hardware, compressive sensing significantly cuts costs, complexity, and measurement times, as it enables the collection of sparse data in real-time, which can then be computationally reconstructed, often utilizing remote servers or cloud computing. This technique has seen success in both imaging^19^ and spectroscopy.^20^

In hyperspectral analysis, it is often assumed that the hyperspectrum embodies a limited array of chemical signatures, making it chemically sparse.^21^ This chemical sparsity allows the hyperspectrum to be represented as a low-rank matrix composed of a finite number of distinct eigenspectra. Consequently, even with sparse sampling, the hyperspectrum can be accurately reconstructed without fidelity loss. This property is particularly relevant to biological data, where many matrices can be deconstructed into lower-rank constituent spectra. Recent advancements in compressive sensing for Raman spectroscopy focus on fast under-sampling schemes and reconstruction via matrix-completion algorithms.^21^ However, the focus of previous studies has predominantly centered on technique development and demonstration rather than utility in practical cases.^21−24^ It is important to note that compressive sensing does not recover data from wavenumber regions where no measurements have been taken in the data set. Also, it does not enhance the resolution of the data through a pretrained model or by applying a super-resolution technique, either in the wavenumber or spatial domain. Compressive sensing works by leveraging the sparsity of the data to reconstruct missing information, but this is only possible within the range of wavenumbers where measurements were performed. In our approach, measurements are taken across a defined wavenumber range, but data from some wavenumbers within this range is randomly missing due to undersampling. Compressive sensing enables us to recover those missing data points based on the information gathered from the available measurements. However, it is essential to understand that compressive sensing does not create new data beyond the measurement range or increase the inherent resolution of the acquired data. It simply reconstructs the missing points from within the measured range by exploiting the structure and sparsity of the signal.

In this work, we explore the practical utility of compressive sensing for spontaneous Raman spectroscopy across various biological samples and data postprocessing. Given the potential benefits of compressive sensing—such as portable hardware, shorter acquisition times, and reduced storage requirements—we investigate applications where these constraints are critical. Examples include skin hydration prediction as a proxy for bioprocess monitoring, cellular studies involving drug molecules, and tissue slice studies, examining both the capabilities and limitations of compressive sensing. The ground truth data set is collected using grating based Raman system at all wavenumbers at all data points. Sparsity is introduced computationally on this data set (Figure 1). This allows us to rigorously assess the accuracy of the signal recovery.

**Figure 1:**
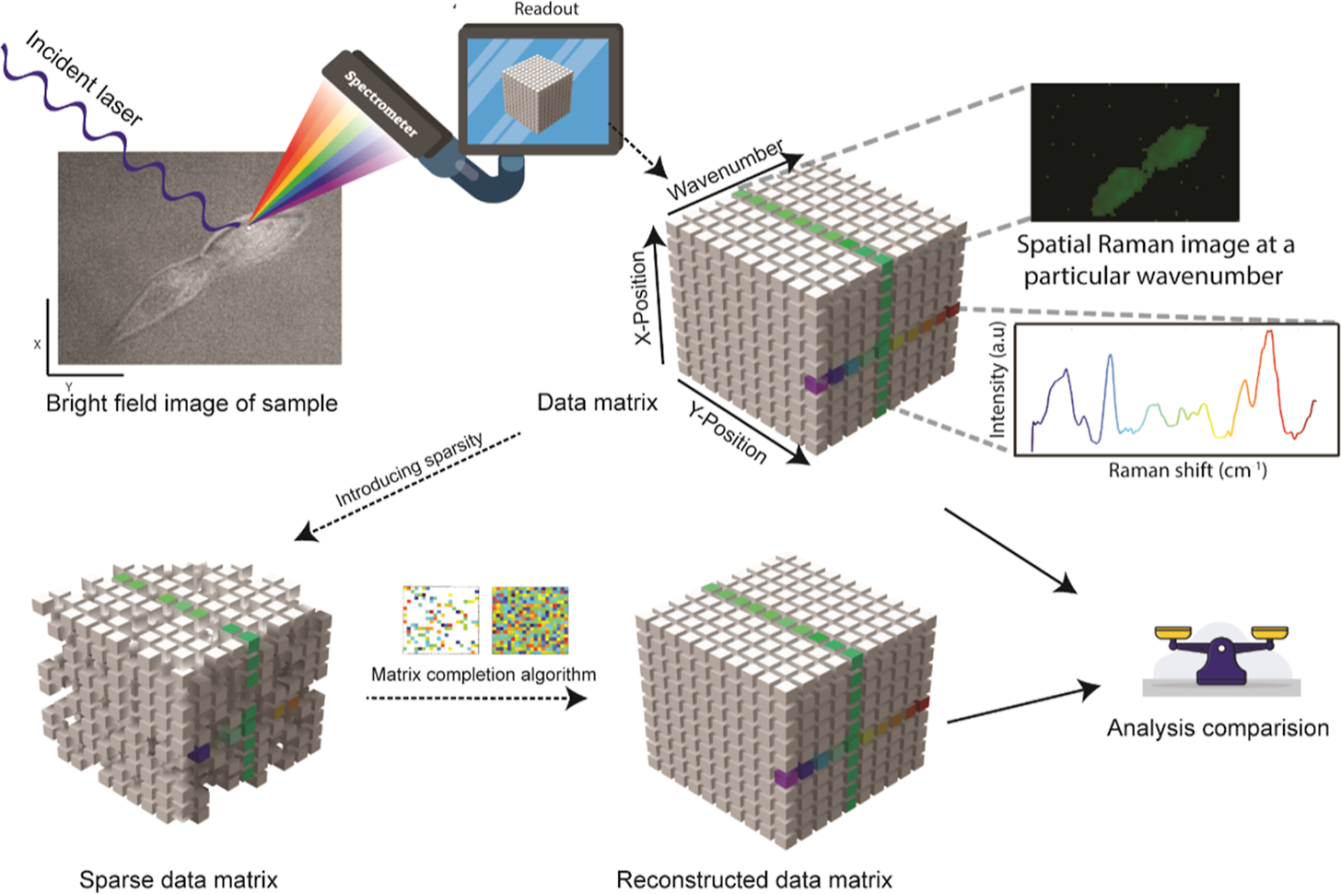
Schematic of the study design. Raw Raman spectra were collected at all possible wavenumbers from a conventional system, giving us a hyperspectral cube. Post processing on the full data set helped us establish the ground truth. Sparsity was introduced in the raw data set at different levels, and the missing points were recovered using the matrix completion algorithm. The final analysis from both data sets was compared.

## Results and Discussions

Unlike other data sets such as brightfield and fluorescence, raw Raman data sets obtained from biological specimens are typically not directly applicable for downstream classification, regression, and peak identification tasks in data analysis pipelines.^1,25^ Raman data must first undergo preprocessing steps before the relevant data analysis task at hand can be effectively carried out. Therefore, relying solely on default reconstruction error calculation is not the most suitable approach to gauge its utility. Instead, comparing the effectiveness of the final task serves as a more pragmatic metric to assess its applicability. Here, we have extensively explored the areas where compressive sensing proves effective and where its efficacy may be limited. The raw Raman data were collected from a confocal Raman microscope (XploRA PLUS, HORIBA Instruments Inc., Edison, NJ, USA), which served as a ground truth for all downstream analyses (Figure 1). To meticulously check the utility of compressive sensing, a varying amount of random sparsity was introduced to the raw Raman data in the spectral domain. The sparsity was introduced using a random choice function in the numpy package in Python, and reconstruction was performed through fast iterative shrinkage-thresholding algorithm (FISTA) [https://github.com/uprestel/Matrix-Completion]. A short technical overview on compressive sensing is presented in the Supporting Information (Section S1).

### Compressive Sensing for Concentration Predictions of Biomolecules with Raman Spectroscopy

**Figure 2:**
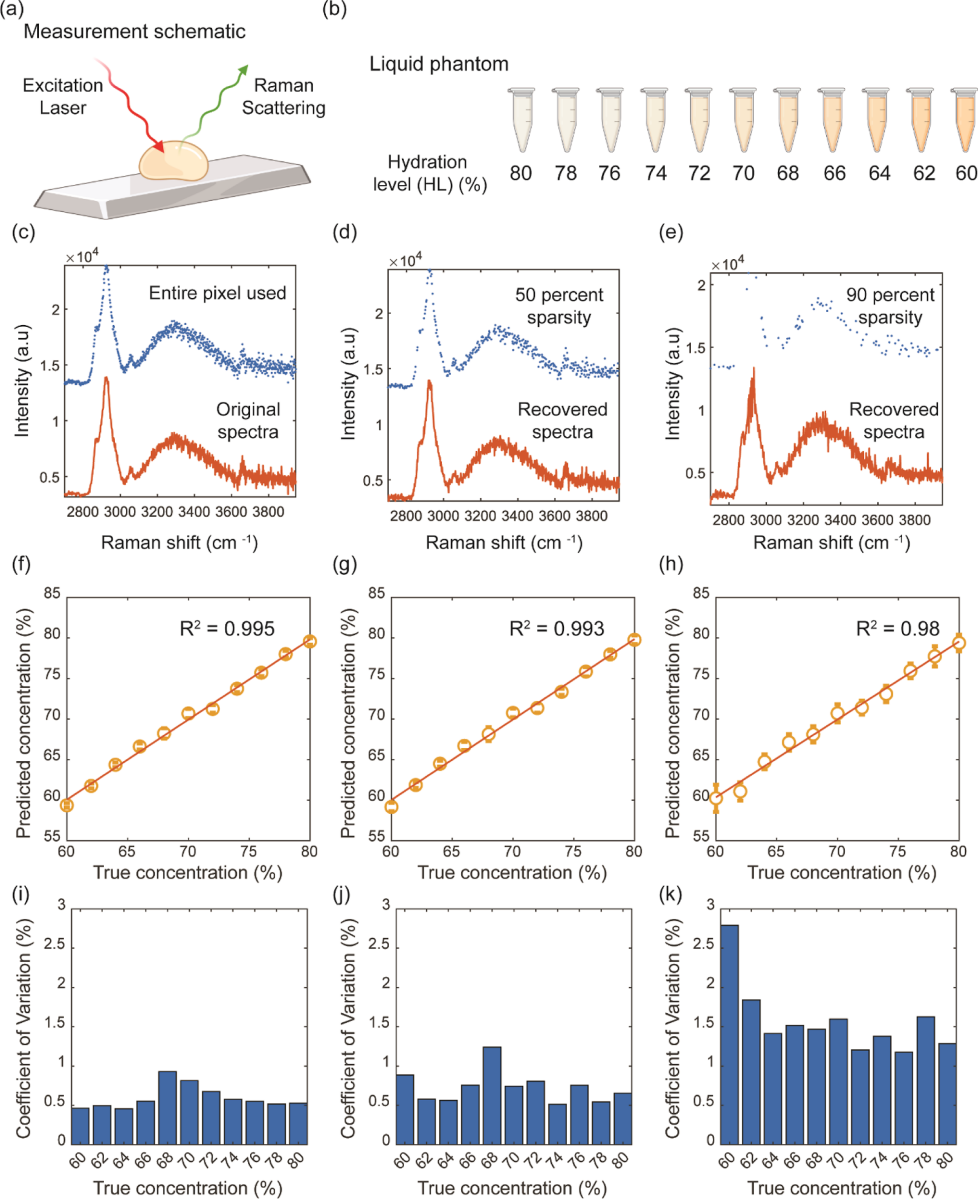
Concentration prediction of biomolecules (a) Measurement schematic for BSA mixed with water. (b) List of different concentrations used for measurement. (c) Scatter plot (blue) and line plot (orange) of raw Raman spectra when the entire pixel was used (0% sparsity). (d) Scatter plot (blue) and recovered Raman spectra (orange) when 50% of pixels (50% sparsity) were used. (e) Scatter plot (blue) and recovered Raman spectra (orange) when 10% of pixels (90% sparsity) were used. Regression plot following partial least-square analysis with leave one concentration out scheme when (f) entire pixel, (g) 50% sparsity, and (h) 90% sparsity was used. Coefficient of variation following partial least-squares prediction of the unknown sample when (i) entire pixel, (j) 50% sparsity, and (k) 90% sparsity was used.

Raman spectroscopy has found many applications in numerous domains ranging from pharmaceuticals^9^ and biotechnology^26^ to environmental monitoring^27^ and industrial processes^28^ for its ability to specifically predict the concentration of constituents^29^ in a label-free manner. For a lot of production units, Raman spectroscopy combined with chemometrics is a workhorse for in-line process monitoring, which falls under the wider umbrella of process analytical technologies (PATs).^30^ A common thread that unites all these applications is the Raman measurement of the sample (Figure 2a) and the use of regression analysis on acquired data.

In this work, we used a simple system of bovine serum albumin (BSA) mixed with water at different concentrations to show applicability of compressive sensing for concentration prediction. Such model systems have been extensively used for skin hydration studies,^31−33^ and they act as a good proxy for any task of concentration prediction of known mixtures. BSA and water were added by weight, and hydration level was calculated as the percentage of water’s weight contribution to the total weight. The concentration range was kept between 80% and 60% (Figure 2b) as any concentration below 60% was not properly miscible, and concentrations higher than 80% were not physiologically relevant for skin hydration studies. We have plotted sparse spectra and recovered spectra with varying levels of sparsity in Figure 2c–e. For the regression analysis, we used the partial least-squares (PLS) method with a leave-one-sample-out scheme. The leave-one-sample-out scheme ensured that each sample was being blind-tested by the trained model, as none of the spectra from that sample made it to the training data. When the entire data set was used, we obtained an *R*^2^ of 0.995 with the highest coefficient of variation as 1% (Figure 2f,i). When 50% of the data were missing at random, the analysis on recovered spectra was able to reach an *R*^2^ of 0.993, with the highest coefficient of variation around 1.4% (Figure 2g,j). Finally, for sparsity as high as 90%, we measured an *R*^2^ of 0.98 with the highest coefficient of variation of around 2.7% (Figure 2h,k). The limit of quantification (LoQ) is defined as a prediction value that can be reliably measured, keeping the concentration coefficient of variation less than 2%, corresponding to the concentration intervals used in our measurements. Using this metric, we determined that for the full data set, the LoQ is 60%. When utilizing a 50% sparse data set, the LoQ remains unchanged at 60%. However, for a 90% sparse data set, the LoQ slightly increases to 62%. The coefficient of variation increases, and the *R*^2^ value decreases with sparsity, which is expected since we have less information to work with. However, the speed improvement from sparsity is significant compared to the marginal improvement in the regression metrics. A system with 90% sparsity can potentially reduce the data acquisition time by a factor of 9 without much compromise in downstream analysis metrics.

Compressive sensing methods work remarkably well in this case due to alignment of the data set with the constraints essential for the method’s efficacy. The skin hydration phantom is a homogeneous mixture of two components and, therefore, satisfies the condition of chemical sparsity extremely well, devoid of any rare or outlier spectral features within the data set. Skin hydration monitoring tools and process analytical devices rely on sensors that continually seek advancements in hardware miniaturization and cost reduction. Given the effectiveness of compressive sensing methods in this domain, there exists a significant opportunity for leveraging this approach. Compressive sensing offers a promising avenue for achieving accurate and efficient data acquisition while minimizing hardware size and costs. As such, integrating compressive sensing into skin hydration monitoring and process analytical tools presents a compelling opportunity to enhance their performance, accessibility, and affordability, thereby benefiting both users and manufacturers alike.

### Compressive Sensing for Studying Cells with Raman Spectroscopy

Cells form the foundational elements of all living organisms, serving as the basic units from which life is built. Consequently, any discourse on the biological applications of Raman spectroscopy must inherently encompass the significant role of Raman imaging in studying these microscopic units. With the improvement in optical technologies, Raman spectroscopy has emerged as a powerful and noninvasive technique for studying the molecular composition and dynamics within cells.^5^ Researchers utilize it to monitor changes in cellular metabolism,^34^ identify different cell types,^35^ assess the distribution of biomolecules within cells,^36^ and investigate the effects of drugs at a molecular level.^37^ However, owing to the weak effect of Raman spectroscopy, collecting spectra from cells takes an excruciatingly long time. A confocal scanning system requires approximately 5 s to capture the data for a single pixel in cell imaging. Consequently, to compile a low-resolution image consisting of 100 by 100 pixels, the process can extend to a duration of around 13 h. This has prohibited large-scale cellular imaging studies with spontaneous Raman spectroscopy. In our study, we demonstrate the effectiveness and limitations of compressive sensing in enhancing cellular imaging with Raman spectroscopy.

**Figure 3:**
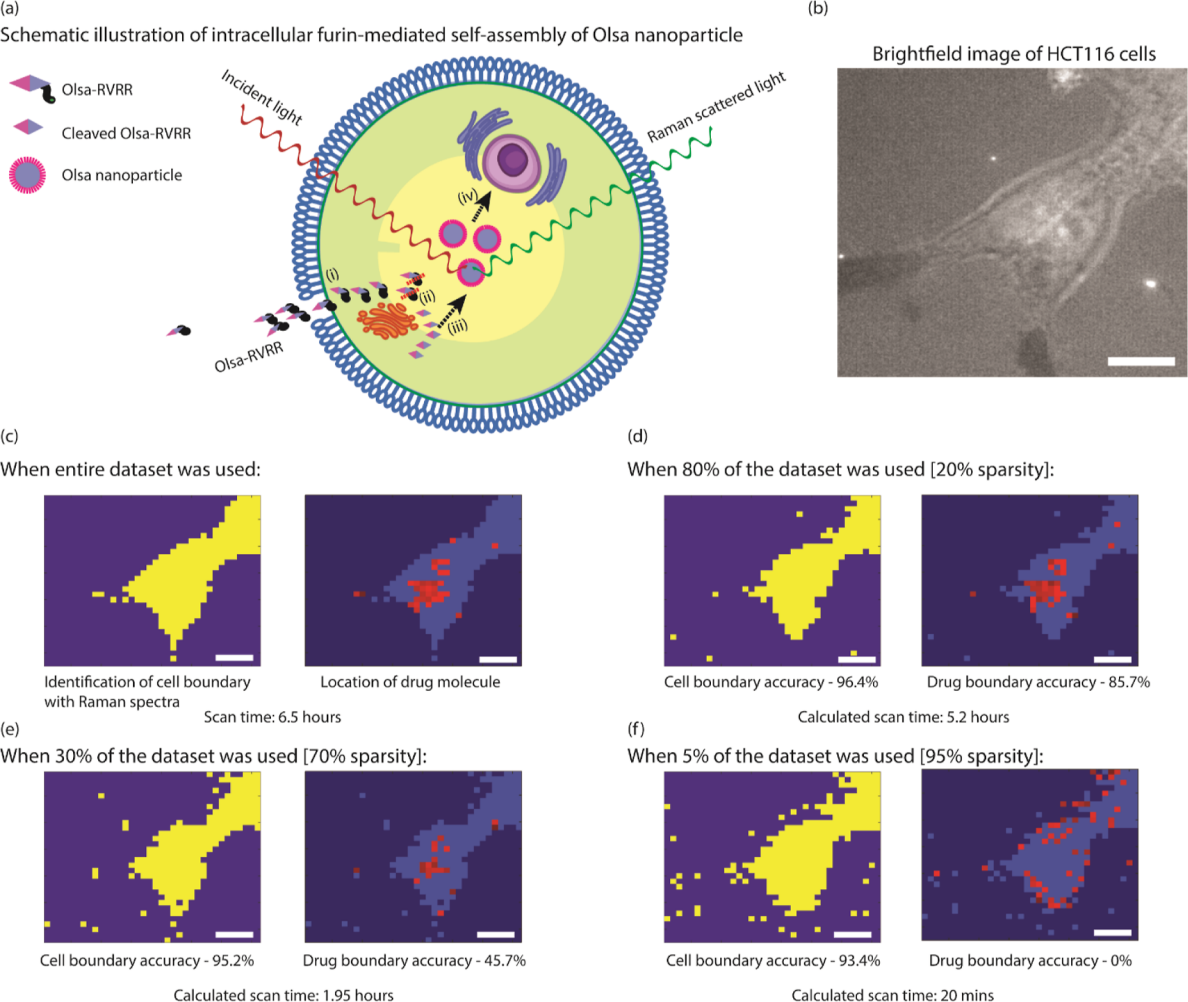
Cell imaging with Raman spectroscopy. (a) Schematic for Raman cell imaging experiment. (i) HCT116 cells are treated with Olsa-RVRR (Raman probe). (ii) Olsa-RVRR probe is cleaved in the presence of furin near the Golgi complex. (iii) A biocompatible click condensation reaction occurs between cleaved products, leading to dimer formation, which subsequently self-assembles into Olsa nanoparticles. The self-assembled nanoparticles give distinct differential signals in Raman spectra. (iv) The intracellular accumulation of Olsa nanoparticles then serves as a reservoir of Olsa molecule inhibiting DNA methylation for tumor therapy. (b) Brightfield image of HCT 116 cells. (Scale bar = 10 μm). (c) Ground truth for cell boundary and pixels with drug (Olsa-RVRR Raman probe) molecule when the entire data set was used (0% sparsity). (d) When 80% of the data set was used, cell boundary accuracy was 96.4%, while drug boundary accuracy was 85.7%. (e) When 30% of the data set was used, cell boundary accuracy was 95.2%, while drug boundary accuracy was 45.7%. (f) When 5% of the data set was used, cell boundary accuracy was 93.4%, while drug boundary accuracy was 0%.

We employed HCT116 cells, which are characterized by their high levels of furin expression.^38^ To target this attribute, we utilized the Olsa-RVRR molecule (Olsa stands for Olsalazine, and RVRR is the cell-penetrating peptide), which undergoes cleavage in the presence of furin.^39^ Upon cleavage, a biocompatible click condensation reaction is initiated between the resultant products, facilitating the formation of dimers. These dimers then proceed to self-assemble into Olsa nanoparticles. The resulting nanoparticles produce unique differential signals observed in Raman spectra, providing a novel approach to cellular imaging of furin-overexpressing cells. A schematic of the entire process can be found in Figure 3a, and a bright field image of the cell is provided in Figure 3b. Raman spectra of the background, cell cytoplasm, and intracellular Olsa nanoparticle are provided in Section S3 of Supporting Information. We collected Raman spectra from a field of view of 48 by 54 μm with 1.5 μm spacing, resulting in an image of 32 by 36 pixels. To delineate cell boundaries, we applied *k*-means clustering with a partition value of *k* = 2. Additionally, the presence of the drug molecule, i.e., the Olsa nanoparticle was identified based on the peak intensity at 1168 cm^–1^. Since this image was created using all the pixels, it provided the ground truth (Figure 3c) to test the accuracy of the compressive sensing framework. In Figure 3, the term “scan time” refers to the actual measurement duration, while “calculated scan time” represents the potential time savings achievable through the use of compressive sensing.

When utilizing 80% of the data, we achieved a cell boundary detection accuracy of 96.4% and a drug molecule localization accuracy of 85.7% (Figure 3d). With 30% of the data, the accuracy for cell boundary and drug molecule localization was 95.2% and 45.7%, respectively (Figure 3e). However, when only 5% of the data set was employed, the cell boundary detection accuracy slightly decreased to 93.4%, while the ability to accurately localize drug molecules dropped to 0% (Figure 3f). The drug molecule is present in about 3% of the total area measured and it can reliably be predicted (>85% accuracy) with around 20% sparsity. As we increase the sparsity in the data, the recovery of spectra which are only present in a few points in the field of view suffers greatly (drug molecule), while the spectra which are in abundance can still be recovered, as is the case with cell cytoplasm spectra. This progressive recovery illustrates the nature of compressive sensing: with minimal data, the algorithm is able to accurately recover the most significant trends in the data set. However, as more information is required to capture finer details, the effectiveness of the reconstruction diminishes with higher sparsity. This finding highlights the trade-off between data reduction and signal fidelity—while compressive sensing efficiently captures dominant features with minimal data, it may struggle to fully reconstruct subtle or complex patterns as sparsity increases.

Even with a substantial portion of the data missing—up to 95%—it is possible to accurately delineate cell boundaries with an accuracy exceeding 90%. This highlights the robustness of compressive sensing techniques applied in processing cell images, demonstrating their ability to extract meaningful information even from significantly sparse data sets. On the other hand, detecting drug molecules poses a greater challenge under conditions of high data sparsity. This difficulty arises because drug molecules typically exist in only trace amounts within the field of view, making it hard for the spectra to be accurately reconstructed from a compressed data set. The challenge of generating unique and precise data from significantly incomplete information, especially for samples present in trace amounts, is a well-known obstacle in the field of matrix completion algorithms.^40^ To the best of our knowledge, this is the first work where we utilize these concepts in an experimental data set specifically to evaluate the degree of sparsity that Raman spectroscopy data can withstand for cell imaging. The principle at play here is that the success of reconstructing the presence and distribution of drug molecules from sparse data sets is inversely proportional to the sparsity of these molecules within the field of view. In essence, the fewer the number of drug molecules present, less sparse the data must be to capture and reconstruct their signal accurately. This nuanced interplay between the sparsity of the data set and the inherent sparsity of the drug molecules in the sample underscores the complexities involved in applying compressive sensing techniques to the detection and imaging of trace substances within cellular environments.

### Compressive Sensing for Studying Tissues with Raman Spectroscopy

Tissue imaging plays a crucial role in biology and medical science, providing invaluable insights into the structural and functional intricacies of living organisms.^41^ It enables researchers and clinicians to visualize and analyze the microscopic architecture of tissues, facilitating the understanding of cellular relationships, disease pathologies, and the effects of therapeutic interventions. This deepened understanding is pivotal for advancements in diagnosis, treatment, and the development of novel medical technologies. Raman spectroscopy stands out among imaging modalities for its unique ability to provide detailed information on the chemical composition of samples without the need for labels or dyes without causing any damage to the sample. However, like in cellular imaging, the practical application of spontaneous Raman spectroscopy in tissue imaging has often been hindered by the slow speed of scanning.

**Figure 4:**
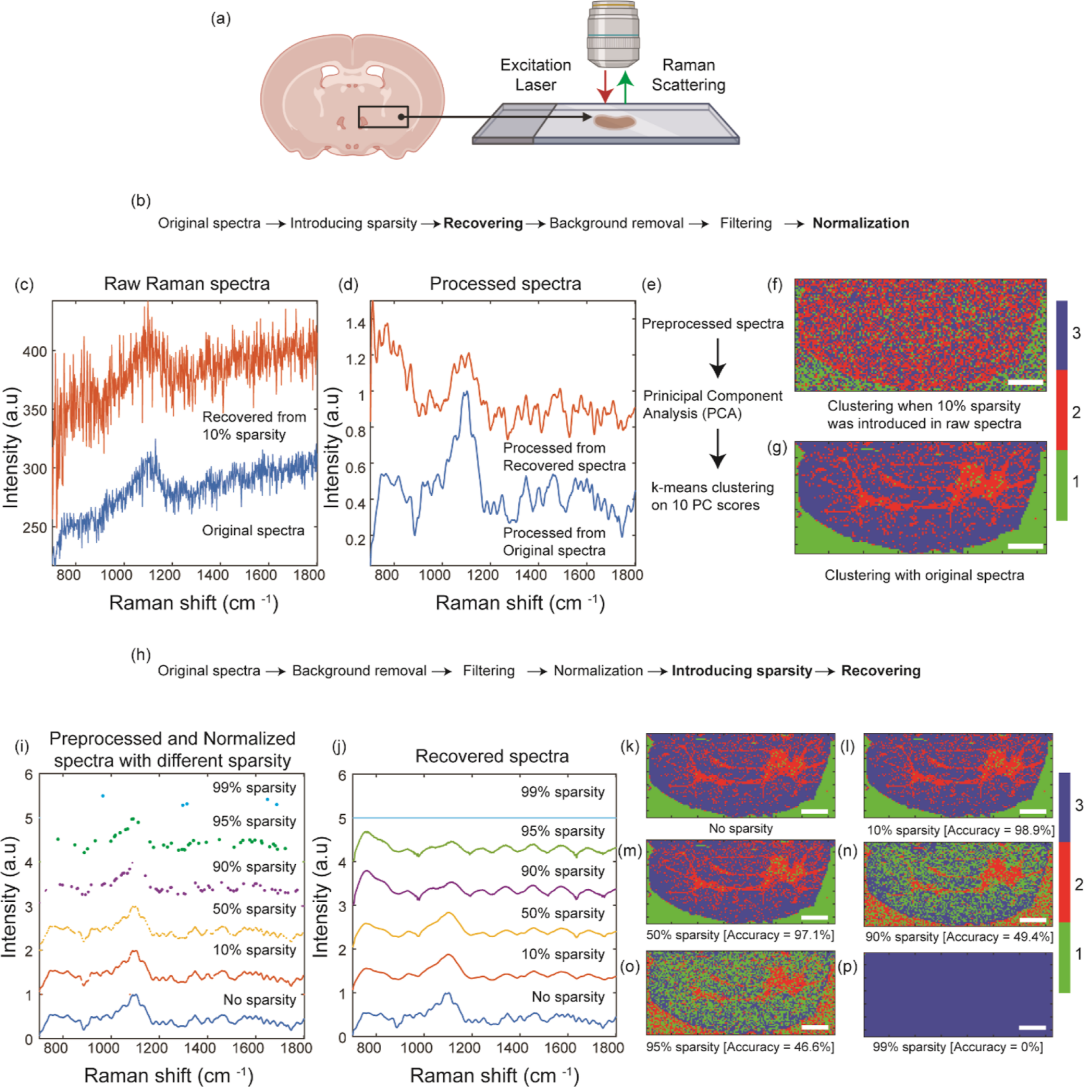
(a) Schematic for tissue imaging with Raman spectroscopy. (b) Spectral processing pipeline for generating (d). (c) Raw Raman spectra collected from the instrument (blue), and recovered raw spectra from 10% sparsity (orange). (d) Processed spectra from the original (blue) and 10% sparse raw data (orange). (e) Analysis pipeline for clustering spectra. (f) Spatial plots of identified clusters using 10% sparse data. (g) Spatial plot of identified clusters from original spectra. (h) Spectral processing pipeline for generating (j). (i) Preprocessed and normalized spectra with different levels of sparsity. (j) Recovered spectra from the sparse data set in (i). Spatial plot of identified cluster with (k) no sparsity, (l) 10% sparsity, (m) 50% sparsity, (n) 90% sparsity, (o) 95% sparsity and (p) 99% sparsity. (Scale bar −1 mm) The color map shows the cluster number from the k-means cluster analysis.

In our study, we conducted Raman spectral scans of fresh-frozen sections of mouse brain (illustrated in Figure 4a), focusing specifically on the cerebellum. The cerebellum is an area of interest due to its distinct anatomical features and the presence of multiple molecular layers, making it an ideal subject for evaluating the capabilities of Raman imaging in revealing complex structures. For the analysis, we utilized the full pixel data from the initial scans (Figure 4c,d) and subjected it to spectral processing (shown in Figure 4b), followed by principal component analysis (PCA). Using the first 10 principal component (PC) scores, we applied *k*-means clustering with *k* = 3 (Figure 4e,g) to differentiate between the background (colored green), gray matter (colored blue), and white matter (colored red) in the brain tissue. However, when we introduced a sparsity of just 10% to the original raw spectra (Figure 4c,d), attempting to mimic conditions of incomplete data, we found that it was impossible to obtain any meaningful clustering (Figure 4f) compared to structures present in clustering with original spectra (Figure 4g). The spectrum provided in Figure 4c,d,i,j correspond to the spectra that would be in the blue region of Figure 4g.

In our prior experiments, including the one involving the detection of drug molecules present in trace amounts, we found that data with up to 20% sparsity could still be reliably analyzed. Therefore, it was unexpected that a mere 10% sparsity would pose significant challenges in our current study. Motivated by this discrepancy, we delved deeper into the issue by applying sparsity to the processed spectra (as shown in Figure 4i,j). These spectra had undergone noise reduction and lacked the fluorescence background that was present in the raw data (Figure 4h), making it cleaner and more conducive to analysis. Remarkably, when working with the processed, cleaner spectra, we were able to reconstruct the morphological structure of the tissue (Figure 4k) with high accuracy, even at varying levels of sparsity. Specifically, we achieved 98.9% accuracy with 10% sparsity (Figure 4l), 97.1% accuracy with 50% sparsity (Figure 4m), and still notable levels of accuracy at higher sparsity levels: 49.4% for 90% sparsity (Figure 4n), 46.6% for 95% sparsity (Figure 4o), plummeting to 0% accuracy at 99% sparsity (Figure 4p). This improvement in reconstruction from cleaner spectra underscores an important insight: when the initial data is less noisy and free from large fluorescence background, the matrix completion algorithms are more effective. This is because, in the presence of excessive noise, small Raman peaks, which are critical for identifying specific features, may be obscured by the overwhelming fluorescence background and dismissed as noise by the algorithm. Essentially, the algorithm then primarily reconstructs the broad fluorescence background, overlooking the subtle yet crucial Raman peaks. The PC loading plot for all the PCA is plotted in Section S2 of the Supporting Information. In Figure S1, the PC component from the original spectra is plotted. In Figure S2, we have plotted the PC component plot for the case where 10% sparsity was introduced to the raw spectra (Figure 4f). We find that the similarities between Figures S1 and S2 are minimal, further confirming that most of the information is lost in the reconstructed spectra. However, in cases where sparsity was introduced in the normalized spectra, we see that the first 7 PC component plots are identical between Figures S3 and S1 for 10% sparsity, the first 5 component plots are identical between Figures S4 and S1 for 50% sparsity, the first 2 component plots are identical between Figures S5 and S1 for 90% sparsity and finally the first component is similar between Figures S6 and S1 for 95% sparsity. At low levels of sparsity (e.g., 10%), the algorithm captures the most dominant patterns in the data, which are represented by the first few PCs. These initial components typically account for the largest variance, reflecting the key structural or spectral features of the data set. As sparsity increases, the available information becomes more limited, leading to a gradual loss of the ability to reconstruct less dominant PCs. These lower-ranked components represent finer details or smaller variance features, which are more sensitive to data reduction and are therefore more difficult to recover with increasing sparsity.

This observation highlights a fundamental limitation of compressive sensing in the context of tissue imaging. To ensure successful reconstruction, the collected raw data must have a sufficient signal-to-noise ratio (SNR). This observation underscores the critical importance of meticulous experimental planning, particularly in the selection of signal acquisition parameters, which must be carefully tailored to the specific characteristics of the sample under investigation. This may include selecting an optimal laser wavelength to mitigate autofluorescence or extending the signal acquisition duration to ensure adequate collection of Raman photons.

## Conclusions

In conclusion, our exploration across diverse applications—from concentration prediction to cell and tissue studies—highlights the nuanced balance between the speed of data acquisition and the fidelity of the resulting analysis when employing compressive sensing techniques in Raman spectroscopy. For concentration prediction of known components, such as in the case of bioprocess monitoring, there is a clear opportunity to produce a huge advantage in speed with only a modest compromise in regression metrics. In cellular studies where the data matrix aligns with the prerequisites for compressive sensing (low-rank assumption), there is only a minimal impact on the analysis metrics. However, for components which are sparsely present in the sample, the reconstruction suffers depending on the level of sparsity during the signal acquisition. For tissue imaging, our findings underscore the importance of the initial data quality. Cleaner spectra, free from large fluorescence backgrounds and excessive noise, allow matrix completion algorithms to perform optimally, demonstrating that the clarity of small Raman peaks is essential for accurate feature identification. The success of compressive sensing hinges not only on the mathematical robustness of the technique but also on a deep understanding of the sample’s characteristics and the experimental setup. As such, the advancements in compressive sensing offer a promising avenue for refining Raman spectroscopic analysis across a spectrum of biological applications, driving forward the capabilities of this technology in both research and clinical settings.

## Materials and Methods

### Skin Hydration Phantom Study

Bovine serum albumin (Sigma-Aldrich A9418) was mixed with HPLC water to create several solutions of different concentrations. These were then dropped on an Aluminum plate and Raman measurements were carried out using a confocal Raman microscope (XploRA PLUS, HORIBA Instruments Inc., Edison, NJ, USA) with a 10× objective lens and 600 grooves/mm grating. The laser wavelength used was 785 nm with 5 s acquisition time and 3 accumulation. The power at the sample spot was 4.3 μW. The software used to control the instrumentation was LabSpec 4.

### Cellular Study

All commercially available materials were obtained from Sigma-Aldrich or Thermo Fisher and used without further purification, unless otherwise specified. All chemicals were reagent grade or better. Olsalazine sodium (Olsa) was purchased from Santa Cruz Biotechnology, and it was conjugated to the cell-penetrating peptide RVRR to create Olsa-RVRR.^38,39^ Furin was purchased from New England Biolabs (2000 U mL^–1^). Milli-Q water (18.2 MΩ·cm) was used throughout all experiments. HCT116 (high-furin expressing) cells (CCL-247, ATCC) were cultured in McCoy’s 5A (modified) medium supplemented with 10% fetal bovine serum and 1% penicillin/streptomycin. CCD-18Co (furin-negative) cells (CRL-1459, ATCC) were cultured in EMEM medium supplemented with 10% fetal bovine serum and 1% penicillin/streptomycin. Cells were cultured at 37 °C and 5% CO_2_ in a humidified atmosphere.

1 ×10^5^ HCT116 were cultured in six-well plates with quartz slides for 24 h. After incubation with 100 μM Olsa-RVRR for 3 h at 37 °C, cells were washed three times with PBS, fixed with 4% paraformaldehyde for 10 min, and washed three times again. Raman spectra were recorded using the XploRA PLUS Raman microscope with a motorized stage and 1200 grooves/mm grating. A 785 nm laser and a 60× water immersion objective were used for sample excitation. For each spectrum, the acquisition time was 10 s with 2 accumulations. The power at the sample spot was 3.2 μW. The spatial resolution for the Raman mapping was 1.5 μm with an image size of 32 by 36 pixels. The software used to control the instrumentation was LabSpec 4.

### Tissue Study

All animal experiments were approved by the Institutional Animal Care and Use Committee (IACUC) of the Johns Hopkins University School of Medicine, which is fully accredited by the American Association for the Accreditation of Laboratory Animal Care (AAALAC). Adult athymic nude nu/nu mice (Taconic Biosciences, Rensselaer, NY) of about three months of age were sacrificed, and brains were frozen in liquid nitrogen vapors. These frozen organs were stored at −80 °C until use. It was then cryo-sectioned using a Leica CM1860 UV cryostat (Leica Biosystems, Wetzlar, Germany) at 10 μm thickness and thaw-mounted onto indium–tin-oxide (ITO) coated slides.^42^ Raman spectra were measured using an XploRA PLUS Raman microscope equipped with a laser wavelength of 532 nm and 1800 grooves/mm grating. The spectra were collected with a 10× objective lens with a 50 μm spatial resolution and an image size of 65 by 145 pixels. For each spectrum, the acquisition time was 0.1 s with 3 accumulations. The power at the sample spot was 50 mW. The software used to control the instrumentation was LabSpec 4.

### Raman Preprocessing Steps

The raw Raman spectra were initially processed using a best-fit polynomial to remove the fluorescence background. A median filtering algorithm was then applied to eliminate spikes, followed by the Savitzky–Golay filter for smoothing. Finally, the spectra were normalized to the highest value in each spectrum.

### Data Analysis

#### Partial Least-Squares

Partial least squares (PLS) is a widely used statistical method in Raman spectroscopy for building predictive models when dealing with complex, multivariate data sets. It is particularly effective in scenarios where the number of variables (wavenumbers) is large and the data is highly collinear, which is often the case in Raman spectra. In this work, we have used the PLS regression model to predict the concentration of skin hydration samples.

In Raman spectroscopy, the spectral data contains a vast number of wavenumbers that correspond to different vibrational modes of the sample’s molecular components. However, many of these wavenumbers are correlated or may not contribute significantly to the specific property or concentration of interest. PLS helps in reducing this complexity by projecting both the spectral data (*X*) and the target variable (*Y*, which could be a chemical concentration, physical property, etc.) into a new latent space that maximizes the covariance between *X* and *Y*. PLS extracts a smaller number of latent variables (components) from the original spectral data that capture the most important variance related to the prediction target.

In this work, we implemented the leave-one concentration-out method for testing, where we left all the spectra belonging to a particular concentration out and trained the PLS model on the remaining spectra. This ensures robustness by avoiding information leakage into the training model. This process was done iteratively over all the concentrations to get the final prediction values for each spectra. The PLS algorithm was implemented using plsregress function in MATLAB. The matrix “beta” of coefficient estimates for the PLS regression model was then used to predict the concentration. Coefficient of variation is calculated by taking the standard deviation of the predicted concentration and dividing it by the mean of the prediction. To report the value in percentage, we multiply it by 100.

#### Principal Component Analysis

Principal component analysis (PCA) is a dimensionality reduction technique commonly used in Raman spectroscopy to simplify complex spectral data while preserving the most important information. Given the high dimensionality and potential redundancy of Raman spectra, PCA is a powerful tool for uncovering patterns, identifying spectral differences, and visualizing relationships within the data.

PCA reduces the number of variables in Raman spectra by creating a new set of variables (PCs) that are linear combinations of the original wavenumbers. The PCs are ranked based on the amount of variance they explain, allowing researchers to focus on just the top components without losing significant information. By analyzing the scores and loadings of the PCs, PCA helps identify patterns, clusters, and outliers in the Raman data, which is useful for distinguishing between different sample types or conditions. PCA is an unsupervised method, meaning it does not require prior knowledge about the data. This makes it particularly useful for exploratory analysis, where the goal is to discover hidden patterns or groupings in Raman spectra.

In this work, we implemented PCA on a tissue data set comprised of ∼9000 spectra using the pca function in MATLAB. The PC scores was further used for *k*-means clustering.

#### *k*-Means

*K*-means clustering is a popular unsupervised machine learning algorithm used to group data points into clusters based on similarity. In the context of Raman spectroscopy, it is frequently applied to analyze and interpret large spectral data sets, especially when the goal is to identify regions or groups of spectra with similar chemical compositions or structural characteristics. *K*-means clustering works by partitioning the data into *K* clusters, where *K* is a user-specified number of clusters. Each data point (in this case, a Raman spectrum) is assigned to one of the clusters based on its similarity to the cluster center, known as the centroid. The algorithm follows these steps:

Initialization: Select *K* initial centroids randomly from the data set.

Assignment step: Assign each data point (spectrum) to the nearest centroid based on a distance metric, typically Euclidean distance.

Update step: Recalculate the centroids by taking the mean of all data points assigned to each cluster.

Repeat: Steps 2 and 3 are repeated until the centroids no longer change significantly, indicating that the algorithm has converged.

In this work, we implemented *k*-means clustering by using kmeans function in MATLAB on a PC score data set of the top 10 PC components obtained from tissue Raman spectra.

## Data Availability

The code and raw data used to generate the results for this paper are freely available on Figshare (https://doi.org/10.6084/m9.figshare.27682647.v1).

## Supporting Information Available

. A technical overview of compressive sensing for spectral measurements, Loading plots for principal component analysis for tissue measurements. Representative spectra from cell measurements (PDF)
