# Supplementary material for: To Acquire or Not to Acquire: Evaluating Compressive Sensing for Raman Spectroscopy in Biology
Source: ACS Sens. 2024 Dec 20;10(1):175–84. doi: 10.1021/acssensors.4c01732 (PMC11773570; doi:10.1021/acssensors.4c01732)
Supplement: Supplementary file 1 — se4c01732_si_001.pdf [file se4c01732_si_001.pdf]

# To acquire or not to acquire: Evaluating compressive sensing for Raman spectroscopy in biology

Piyush Raj[1], Lintong Wu[1], Jeong Hee Kim[1], Raj Bhatt[2], Kristine Glunde[3,4,5], Ishan Barman[1,3,5*]

1. Department of Mechanical Engineering, Johns Hopkins University, Baltimore, Maryland 21218, USA
2. Hackensack Meridian School of Medicine, Nutley, New Jersey 07110, USA
3. The Russell H. Morgan Department of Radiology and Radiological Science, The Johns Hopkins University, School of Medicine, Baltimore, Maryland 21205, USA
4. Department of Biological Chemistry, The Johns Hopkins University School of Medicine, Baltimore, Maryland 21205, USA
5. The Sidney Kimmel Comprehensive Cancer Center, The Johns Hopkins University School of Medicine, Baltimore, Maryland 21287, USA

*Corresponding author: Ishan Barman – ibarman@jhu.edu

## Supplementary Information

### 1. Compressive Sensing for Spectral Measurements: A Technical Overview

Compressive Sensing (CS) is a signal processing technique that exploits the sparsity of data in a given domain to recover high-dimensional signals from a reduced number of measurements. In spectral measurements, CS enables efficient data acquisition by significantly undersampling the signal while still allowing accurate reconstruction. This method is particularly advantageous for applications requiring high-throughput or time-sensitive spectral data acquisition, such as Raman or infrared spectroscopy.

**Fundamentals of Compressive Sensing**

A signal $x \in R^n$ can be described as sparse or compressible if it has a concise representation in a transform domain. For instance, a Raman spectrum may have most of its energy concentrated in a few coefficients when represented in a basis such as wavelets or the Fourier domain. The key principle in compressive sensing is to project the high-dimensional signal x onto a lower-dimensional measurement space using a sensing matrix $\Phi \in R^{m \times n}$ (where $m \ll n$) This results in a measurement vector $y = \Phi x$, containing fewer measurements than the original signal.

Given that the signal is sparse in some basis $\Psi$, the recovery problem becomes an optimization task:

$$\hat{x} = \arg\min \|x\|_1 \quad \text{subject to} \quad y = \Phi\Psi x.$$

Here, $\|x\|_1$ represents the L1-norm, which encourages sparsity in the recovered signal, and $\Phi\Psi$ is the measurement matrix capturing the transformation between the original signal and its sparse representation.

**Recovery Methods in Compressive Sensing**

(a) **Basis Pursuit (BP):** BP solves the $L_1$ minimization problem exactly by seeking the sparsest signal x that fits the observed measurements y. While BP provides high-quality signal recovery, its computational cost increases with the dimensionality of the data, making it less suitable for real-time applications involving large spectral datasets.

**(b) Orthogonal Matching Pursuit (OMP):** OMP is a greedy algorithm that iteratively selects the dictionary elements (columns of $\Psi$) most correlated with the current residual. OMP is computationally faster than BP, but it performs best when the signal is strictly sparse and may degrade with compressible signals or high noise levels.

(c) **Iterative Shrinkage-Thresholding Algorithms (ISTA):** ISTA is an iterative method that addresses the $L_1$ regularized minimization problem. It iteratively updates the solution using gradient descent followed by a shrinkage (or soft thresholding) operation to enforce sparsity.

**(d) Douglas-Rachford Splitting (DRS):** Douglas-Rachford Splitting (DRS) is a powerful iterative algorithm designed to solve optimization problems involving the sum of two convex functions. In the compressive sensing context, DRS is applied to handle the minimization of the following objective:

$$\hat{x} = \arg\min \lambda \|x\|_1 + \frac{1}{2}\|y - \Phi x\|_2^2,$$

where the $L_2$ norm enforces data fidelity, and the $L_1$ norm encourages sparsity.

- Mechanism: DRS splits the original problem into two sub-problems: one focused on enforcing the sparsity constraint via the $L_1$ norm and another on maintaining data fidelity via the $L_2$ norm. The algorithm iterates between these two proximal operators, progressively refining the solution until convergence.
- Advantages: DRS handles large-scale problems efficiently by breaking them into simpler sub-tasks. It is particularly effective for spectral recovery due to its fast convergence properties and ability to work with non-differentiable terms like the $L_1$ norm.

(e) **Fast Iterative Shrinkage-Thresholding Algorithm (FISTA):** FISTA is an accelerated variant of ISTA that dramatically improves the convergence speed by incorporating a momentum term. It solves the same sparse recovery problem but with significantly fewer iterations compared to standard ISTA.

- Mathematical Formulation: FISTA updates the solution $x_k$ at each iteration using:

$$x_{k+1} = \text{soft}(z_k - \mu \nabla f(z_k), \lambda\mu),$$

Where $z_k$ is a momentum term, $\mu$ is the step size, and $\text{soft}(\cdot)$ denotes the soft thresholding operator that enforces sparsity. The momentum term $z_k$ is updated based on both the current and previous estimates to accelerate convergence.

- Advantages: FISTA reduces the computational time by incorporating acceleration, making it well-suited for real-time or large-scale spectral recovery applications. It balances computational efficiency and accurate reconstruction, making it a preferred method in compressive sensing for spectroscopy.

## 2. Principal Component Loading Plots

**PC loading plot for original tissue spectra**

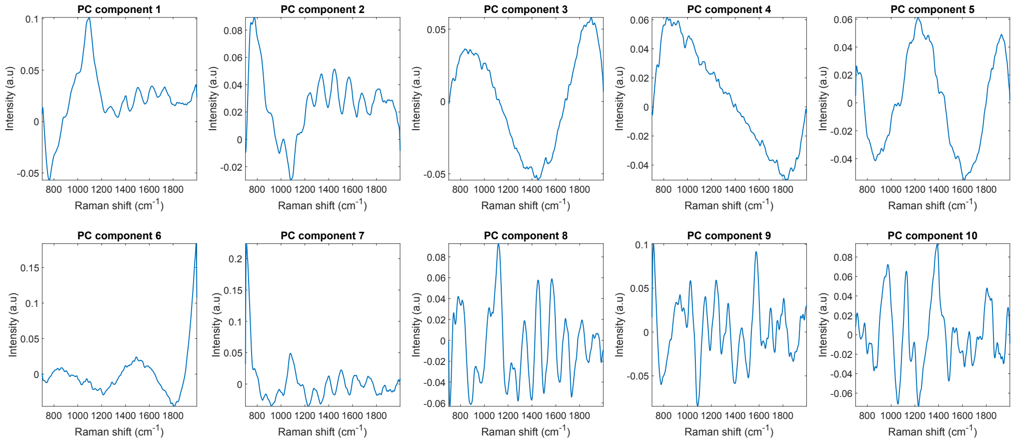


**Figure S1:** PC component loading plot for the case where original spectra was processed without any sparsity

**PC loading plot for the case where 10% sparsity was introduced to original raw tissue spectra**

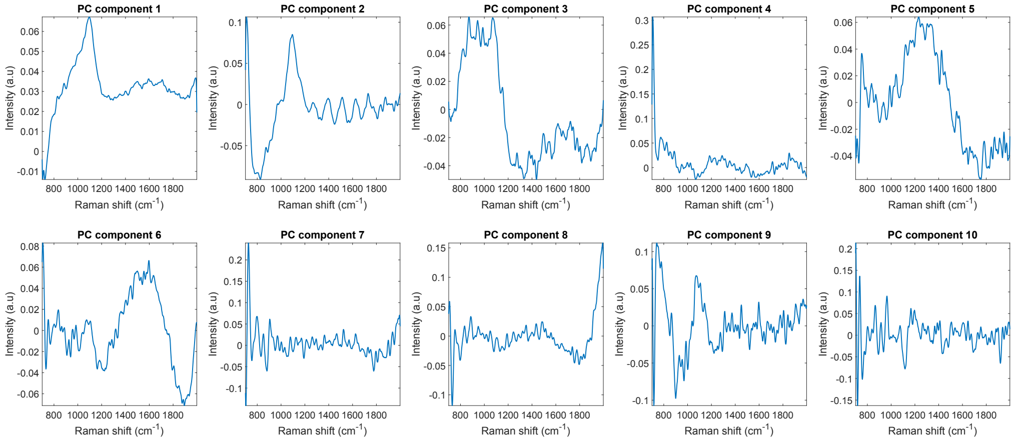


**Figure S2:** PC component loading plot for the case where 10% sparsity was introduced to the original raw tissue spectra. The raw tissue spectra were attempted to recover using compressive sensing algorithm. The entire pipeline mentioned in Figure 4b was used to get the processed spectra and then principal component analysis was performed.

**PC loading plot for the case where 10% sparsity was introduced to normalized raw tissue spectra**

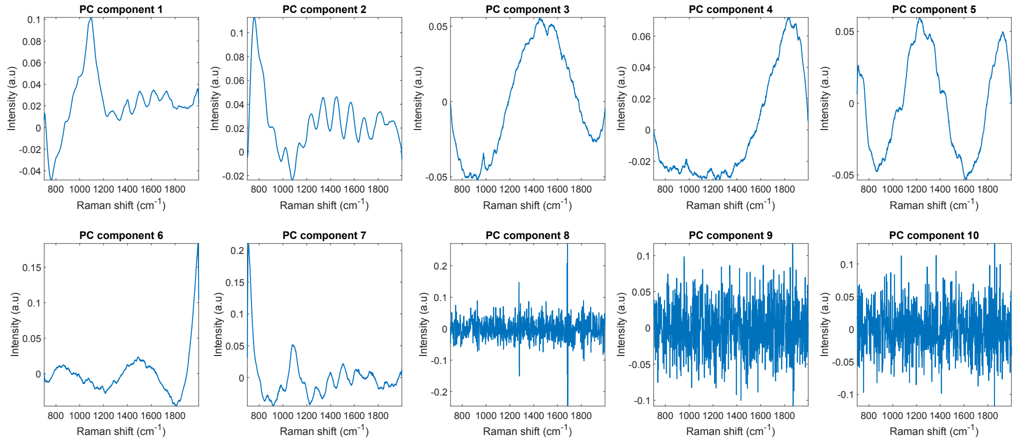


**Figure S3:** PC component loading plot for the case where 10% sparsity was introduced to the normalized tissue spectra. The entire pipeline mentioned in Figure 4h was used to get the processed spectra, and then principal component analysis was performed.

**PC loading plot for the case where 50% sparsity was introduced to normalized raw tissue spectra**

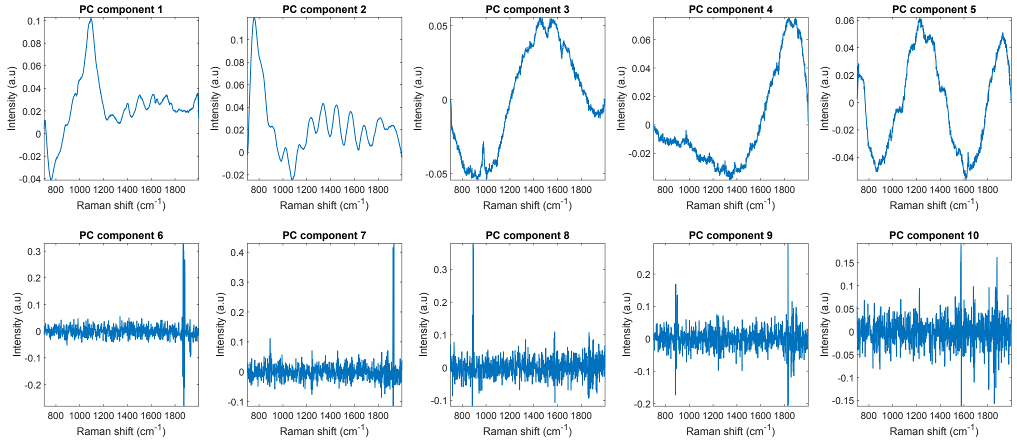


**Figure S4:** PC component loading plot for the case where 50% sparsity was introduced to the normalized tissue spectra. The entire pipeline mentioned in Figure 4h was used to get the processed spectra, and then principal component analysis was performed.

**PC loading plot for the case where 90% sparsity was introduced to normalized raw tissue spectra**

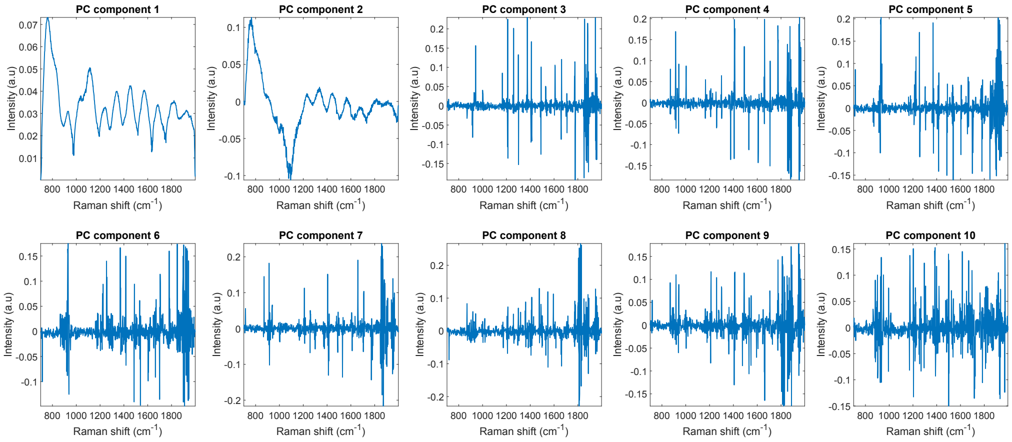


**Figure S5:** PC component loading plot for the case where 90% sparsity was introduced to the normalized tissue spectra. The entire pipeline mentioned in Figure 4h was used to get the processed spectra, and then principal component analysis was performed.

**PC loading plot for the case where 95% sparsity was introduced to normalized raw tissue spectra**

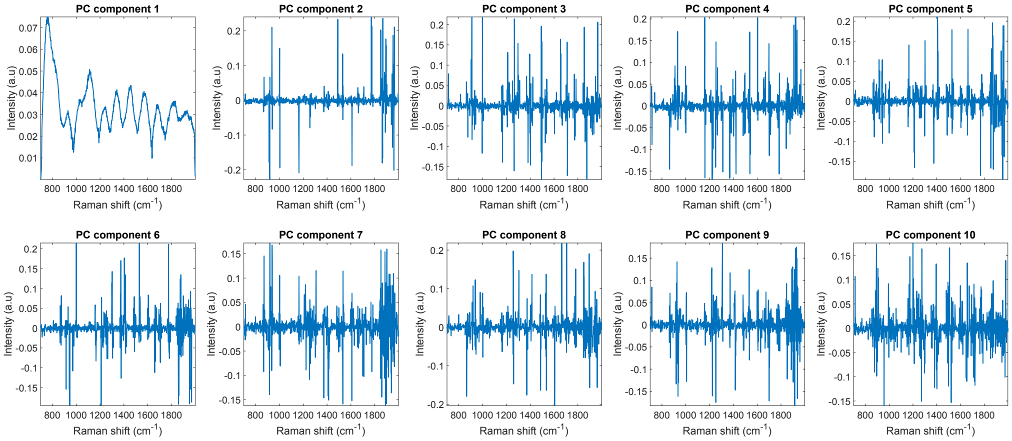


**Figure S6:** PC component loading plot for the case where 95% sparsity was introduced to the normalized tissue spectra. The entire pipeline mentioned in Figure 4h was used to get the processed spectra, and then principal component analysis was performed.

## 3. Spectra from cellular studies

Normalized Raman spectra for spectra collected in cellular studies.

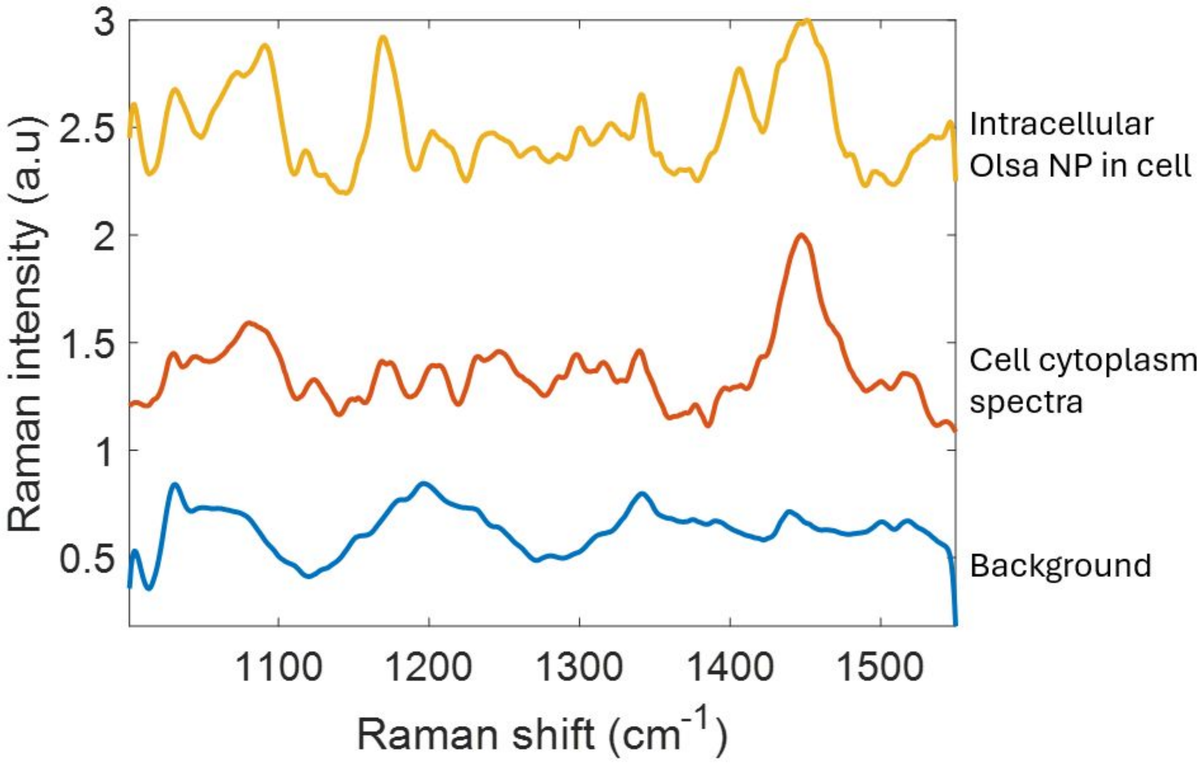


**Figure S7:** Normalized Raman spectra from cellular study: Background spectra (blue), Cell cytoplasm spectra from HCT116 cell line (Red) and Intracellular Olsa nanoparticle in cell (Yellow)
